# Health care seeking behaviour of mothers towards diarrheal disease of children less than 5 years in Mekelle city, North Ethiopia

**DOI:** 10.1186/s13104-018-3850-3

**Published:** 2018-10-22

**Authors:** Tedros Fissehaye, Ashenafi Damte, Atsede Fantahun, Kahsu Gebrekirstos

**Affiliations:** 1 Ayder Comprehensive Referral Hospital, Mekelle University, Mekelle, Ethiopia; 2 Department of Nursing, College of Health Sciences, Mekelle University, Mekelle, Ethiopia

**Keywords:** Health care, Children, Diarrhea, Seeking behavior, Mother

## Abstract

**Objective:**

To assess the health care seeking behavior of mothers on diarrheal disease of under five children and associated factors in Mekelle City, Northern Ethiopia.

**Result:**

This study revealed that 72.5% (n = 58) of the mothers who reported their children had diarrhea had sought health care facilities. Three quarter, (75.9%) of them was seeking health in the public health care facility. Majority, 89.3% of those children who had severe diarrhea sought at health care facilities. In the multivariable analysis, severity of diarrhea (P = 0.04) and blood in stool) were the significantly associated factors with health seeking behavior of mothers for childhood diarrhea.

## Introduction

Diarrhea is defined as having loose or watery stools at least three times a day, or increased frequency of stool as reported by the mother [1]. Although most childhood diarrhea episodes are mild, acute cases can lead to death or other severe consequences as a result of severe fluid loss and dehydration [1].

Globally, childhood diarrhea is among the main killers of children under the age of 5 years [2]. In 2015, 9% of deaths of children under age 5 years are caused by diarrheal disease alone [2]. Most of child deaths from diarrhea occur in the low-income regions of the world in which nearly 90% of the deaths occur in sub-Saharan Africa and South Asia [3]. As a result, child hood diarrhea has long been regarded as a disease of poverty because it is mainly associated with factors such as, under nutrition and lack of access to essential services such as toilets and clean water [4].

Children are dying because services are provided piecemeal and those most at risk are not being reached. For instance, children are not receiving life-saving treatment, and only 35% of children with diarrhea receive oral rehydration therapy [3]. United Nations Children’s Fund (UNICEF) recommended combination of oral rehydration salts (ORS) and zinc supplementation can reduce the severity of diarrhea while preventing relapse and dehydration [4]. Coverage of zinc supplementation for diarrhea treatment is particularly low because introduction and scale-up in most low- and middle-income countries has only occurred recently [2].

Deaths due to these diseases are largely preventable through optimal breastfeeding practices and adequate nutrition, vaccinations, hand washing, safe drinking water and basic sanitation, among other measures. Since 1990, Ethiopia has shown a remarkable reduction in under five mortality and the country is one of the few countries that has achieved the millennium development goal 4 (MDG 4) of reducing child deaths by two thirds [5]. Ending preventable childhood deaths and consequences is possible through proven cost-effective preventive and curative interventions and does not demand advanced technology. Unfortunately, there is a gap in coverage of relevant and effective interventions and has not yet reached the levels required for the desired impact. Improvements in drinking water, sanitation and hygiene are reducing diarrheal infections, but only two in five children in the world get appropriate treatment when they fall ill [2].

Through the support from UNICEF and other partners, the government of Ethiopia has also been working to strengthen the integrated community case management (ICCM) of diarrhea, malaria and pneumonia. Yet, coverage of these treatments is unacceptably low only 2% and 26% of children receive zinc and ORS, respectively. In general, delayed treatment-seeking behavior and low utilization of health services are key bottlenecks to treating children under five [5]. Improving access to treatment for diarrhea can help dramatically reduce under 5 years deaths in Ethiopia. Improving and optimizing care seeking behaviors and thereby increasing utilization of ICCM and IMNCI treatment services for pneumonia and diarrhea [5].

Yet, the knowledge about how and when families seek treatment for these childhood diarrheal illnesses are not well known and utilization of HEWs at the health post for child illness has been found to be very low. Little is known about reasons for low utilization of health care services and care seeking behaviors for this population [6]. Therefore, this study with fill the gap by providing information on mother’s health care seeking behavior and its determinants.

## Main text

### Methods

This community based cross-sectional study was conducted in Mekelle City, Tigray regional state, Northern Ethiopia from March 2015 to June, 2016. All mothers who have under five children in Mekelle city were the source population and selected mothers were the study population. A multi-stage sampling technique was used to select 540 mothers. Four weredas (Districts) were selected using lottery method and ketenes (Sub districts) was selected from the selected wereda randomly and number of households hold under five children was taken from health extension workers and sampling frame was made from it. Then systematic sampling technique was employed for household’s selection from each kebeles. The data was collected using structured interviewer administered questionnaires adapted from the World Health Organization (WHO) generic protocol for a community-based survey on utilization of health care service for gastroenteritis [7]. Four nurses and two B.Sc. nurses were recruited for data collection and supervision respectively. After data collection, each questionnaire was checked for completeness and the data was edited, coded, entered, cleaned and analyzed using SPSS for windows version 20. Descriptive statistics was computed to determine health seeking behavior as well as binary and multiple logistic regression analysis was performed to assess the relationship between dependent and independent variables. The degree of association between independent and dependent variables was assessed using odds ratio with 95% confidence interval or with respective to P-value < 0.05. Efforts were made to assess whether the necessary assumptions for the application of multiple logistic regression were fulfilled. In this regard, in the final model, the Omnibus test of model coefficients had a Chi square value of 23.80 and a probability of P = 0.022, and Hosmer Lemeshow had Chi square value of 12.74 with a significance of 0.121 and therefore the model is good fit model. Finally, the results of study components were presented using texts, graphs and tables.

### Result

#### Socio-demographic characteristics

In this study, a total of 540 mothers whose children aged under five (0–59 months) were participated making the response rate of 100%. The median (IQ range) of age of the mothers was 29 (± 6) years with more than half, 301 (55.70%) of them were found between age range of 20–29 years. Four hundred sixty-six (86.30%) of mothers were Orthodox Christianity by religion. Regarding the educational status of mothers, 483 (89.40%) of them had attended formal education and out of them 179 (33.10%) had accomplished diploma and higher institution. Majority, 517 (95.70%) of mothers were married, 263 (48.7%) house wife by occupation. The average (median) monthly household income of the study participants was 2700 (±2000) Ethiopian Birr, in which one third, 160 (29.60%) of them had monthly income ranged 1001–2000 Ethiopian Birr (Table 1).

**Table 1:** Socio-demographic characteristics of the respondents in Mekelle City, Ethiopia 2016 (n = 540)

Variables	Category	Frequency	Percentage (%)
Age of the child in months	< 6	56	10.4
	6–11	69	12.8
	12–23	133	24.6
	24–59	282	52.2
Mothers’ age in years	≤ 19	1	2
	20–24	58	10.7
	25–29	243	45.0
	30–34	163	30.2
	35–39	62	11.5
	≥ 40	13	2.4
Religion	Orthodox	466	86.3
	Muslim	63	11.7
	Protestant	8	1.5
	Catholic	3	0.6
Marital status	Single	4	0.7
	Married	517	95.7
	Divorced	16	3.0
	Widowed	3	0.6
Educational Status	Formal education	57	10.6
	Elementary school	137	25.4
	Secondary school	167	30.9
	Diploma and above	179	29.1
Occupation of mother	House wife	263	48.7
	Farmer	7	1.3
	Civil servant	121	22.4
	Private	92	17.0
	Student	10	1.9
	Merchant	46	8.5
	Other	1	0.2
Occupation of father	Farmer	10	1.9
	Civil servant	158	29.3
	Private	101	18.7
	Retired	4	0.7
	Trader	146	27.0
	Other^a^	121	22.4
Average monthly income in Ethiopian Birr	< 1000	71	13.1
	1001–2000	160	29.6
	2001–3000	142	26.3
	3001–4000	62	11.5
	4001–5000	55	10.2
	> 5000	50	9.3

^a^Daily laborer, construction, Broker

#### Magnitude and care seeking behavior of childhood diarrhea

From the total 540 under five children, there were 80 (14.81%) children who experienced diarrhea. Out of these 20 (3.7%) of them had a diarrhea of 2 weeks or longer duration. The most common symptoms reported was increased thirst 48 (60.0%) followed by irritability 44 (55.0%), and decreased fluid intake 43 (53.8%). This study found that 58 (72.5%) of mothers were reported seeking care at health care facilities (both public and private). Health center was the common public health facility where mothers took their children to seek health care (Table 2).

**Table 2:** Occurrence and health care seeking behavior of childhood diarrhea in Mekelle City, Tigray, Ethiopia, 2016 (n = 540)

Variables	Category	Frequency	Percent
Presence of diarrhea	Yes	80	14.8
	No	460	85.2
Severity of diarrhea	Mild	18	3.3
	Moderate	34	6.3
	Severe	28	5.2
Duration of diarrhea (weeks)	< 2	20	25
	≥ 2	60	75
Clinical manifestation^a^	Increased thirsty	48	60
	Irritability	44	55
	Decreased fluid intake	43	53
	Lethargic	41	51.3
	Sunken eye	39	48.8
	Blood in stool	8	10
	Others	14	17.5
Measures taken for sickness	Health facilities	58	72.5
	Pharmacy	7	8.8
	Home remedies	6	7.5
	Traditional healer	3	3.8
	No action	2	2.5
	Others	4	5
Type of facility	Public health facility	44	75.9
	Private health facility	14	24.1
Type of public health facility	Health center	34	77.3
	Hospital	10	22.7
ORS given	Yes	69	86.3
	No	11	13.7

^a^More than one answer was possible

This study also revealed that 25 (89.3%) children who had severe diarrhea sought at health facility. Among all children with diarrhea, 69 (86.3%) of them had received oral rehydration salt (ORS) both at home and at health facility. Fifteen (18.8%) of mothers had also reported that as their children were admitted to hospitals.

#### Factors associated with mother’s health seeking behavior

Mother’s health seeking behavior was assessed for its association with Socio demographic, health service, illness characteristics as well as by way of traditional practices. The bivariate analysis model showed that severity of the illness (diarrhea) was significantly associated with the health seeking behavior of mothers (P = 0.008). In the multivariable analysis, adjusting possible confounding variables, severity (P = 0.04) and blood in stool [AOR = 0.13; 95% CI (0.02, 0.88)] were the significantly associated factors with health seeking behavior of mothers for the childhood diarrhea. On the other hand, child age, number of under five children, educational status, monthly household income, availability of transportation, persistency of diarrhea and other characteristics of the illness were not significant factors in this study (Table 3).

**Table 3:** Factors associated with mothers’ health care seeking behavior towards childhood diarrhea in Mekelle city, North Ethiopia 2016

Variables	Seeking structured health care facility			
	No	Yes	COR	AOR
Child age in months				
< 6	3 (42.9%)	4 (57.1%)	0.552 [0.11, 2.85]	–
6–11	1 (16.7%)	5 (83.3%)	2.07 [0.22, 19.63]	–
12–23	6 (23.1%)	20 (76.9%)	1.38 [0.44, 4.29]	–
24–59	12 (29.3%)	29 (70.7%)	1	
Marital status				
Married	20 (26.3%)	56 (73.7%)	1	
Unmarried	2 (50.0%	2 (50.0%)	0.356 [0.047, 2.71]	0.24 [0.01, 11.53]
Occupation				
Housewife	13 (37.1%)	22 (62.9%)	1	
Civil servant	3 (16.7%)	15 (83.3%)	2.96 [0.72, 12.18]	–
Private	6 (23.1%)	20 (76.9%)	1.97 [0.63, 6.17]	–
Other	0 (0.0%)	1 (100.0%)	9.5E8 [0.000, –]	–
Educational status				
Illiterates	2 (25.0%)	6 (75.0%)	1	
Literate	20 (27.8%)	52 (72.2%)	0.867 [0.161, 4.66]	–
Number of under five children				
1 child	13 (26.0%)	37 (74.0%)	1	
2 or more	9 (30.0%)	21 (70.0%)	0.820 [0.30, 2.24]	–
Age of mother				
< 30	12 (26.7%)	33 (73.3%)	1	
30–39	9 (28.1%)	23 (71.9%)	0.93 [0.34, 2.56]	–
≥ 40	1 (33.3%)	2 (66.7%)	0.73 [0.60, 8.77]	–
Sex of the child				
Male	13 (26.5%)	36 (73.5%)	1	
Female	9 (29.0%)	22 (71.0%)	0.883 [0.32, 2.404]	–
Presence of television				
No	3 (60.0%)	2 (40.0%)	1	
Yes	19 (25.3%)	56 (74.7%)	4.421 [0.68, 28.5]	4.96 [0.43, 57.39]
Presence of vehicle (car)				
No	19 (27.1%)	51 (72.9%)	1	
Yes	3 (30.0%)	7 (70.0%)	0.87 [0.204, 3.71]	–
Increased thirst				
No	9 (28.1%)	23 (71.9%)	1	
Yes	13 (27.1%)	35 (72.9%)	1.054 [0.388, 2.86]	–
Irritability				
No	12 (33.3%)	24 (66.7%)	1	
Yes	10 (22.7%)	34 (77.3%)	1.70 [0.63, 4.57]	1.24 [0.27, 5.59]
Decreased fluid intake				
No	13 (35.1%)	24 (64.9%)	1	
Yes	9 (20.9%)	34 (79.1%)	2.05 [0.76, 5.55]	0.73 [0.17, 3.10]
Lethargy				
No	13 (33.3%)	26 (66.7%)	1	
Yes	9 (22.0%)	32 (78.0%)	1.78 [0.66, 4.809]	0.50 [0.10, 2.58]
Sunken eye				
No	14 (34.1%)	27 (65.9%)	1	
Yes	8 (20.5%)	31 (79.5%)	2.009 [0.73, 5.52]	0.55 [0.10, 3.01]
Blood in stool				
No	18 (25.0%)	54 (75.0%)	1	
Yes	4 (50.0%)	4 (50.0%)	0.333 [0.75, 1.47]	0.13 [0.02, 0.88]
Persistency of diarrhea				
No	17 (28.3%)	43 (71.7%)	1	
Yes	5 (25.0%)	15 (75.0%)	1.19 [0.373, 3.77]	–
Severity of diarrhea				
Mild	10 (55.6%)	8 (44.4%)	1	
Moderate	9 (26.5%)	25 (73.5%)	3.47 [1.044, 11.6]	9.55 [1.11, 81.74]
Severe	3 (10.7%)	25 (89.3%)	10.42 [2.29, 47.44]	44.6 [2.23, 893.2]
Previous admission history				
No	20 (30.8%)	45 (69.2%)	1	
Yes	2 (13.3%)	13 (86.7%)	2.89 [0.59, 14.013]	5.41 [0.52, 56.39]
Kind of available transportation				
Foot step	14 (35.0%)	26 (65.0%)	1	
Taxi	6 (20.7%)	23 (79.3%)	2.064 [0.68, 6.26]	1.65 [0.43, 6.40]
Bajaj	2 (18.2%)	9 (81.8%)	2.423 [0.46, 12.79]	1.68 [0.21, 13.20]

### Discussion

This study was primarily aimed to assess the health care seeking behavior of mothers on diarrheal disease of under 5 years children in Mekelle city to help to improve the health care seeking behavior of mothers for the emergence of diarrhea.

In this study, it was found that from 540 of the total households, childhood diarrheal illness was reported by 80 (14.81%) within 01 months recall period. This is similar with period prevalence reported from studies conducted earlier in Mekelle, Tigray Ethiopia (16.4%) [8] And in Urban Slum of Delhi, India which was 14.8% [9]. But it is lower than the period prevalence reported from the studies conducted earlier in West Shoa, Ethiopia (22.1%), Sierra Leone (25.6%), rural Niger (36.8%) and it is higher as compared to the findings from studies conducted in Mirzapur, rural Bangladesh, which was 7.4% [8–13]. The difference in the reported period prevalence of diarrhea might be due to the difference in geographic, seasonal variation and socioeconomic conditions.

This study revealed that 72.5% (n = 58) of the mothers who reported their children had diarrhea had sought health care facilities. More or less, similar report was made by studies carried out in West Shoa, Ethiopia (69.3%), Central Ethiopia (77.0%) and Rural Niger (70.4%). But it is lower that study findings from Serra Leone, which was 85% [11–14]. This study also showed that 89.5% of those children who had severe diarrhea had sought the health facility which is similar with the findings done from rural Niger, which was 83.8% [12]. This might be an indicator of as mothers are being heath seeker while their child is seriously ill.

This study found that all mothers 100% (n = 58) who reported that they sought health care facilities have taken their children to the health facilities within 1–72 h which is higher than the result finding from west Shoa, Ethiopia, which was 83.2% [13]. This difference might be due to the reason that these mothers were good in seeking of health on perspective of time because most of them tended to act and brought their sick children with in short period of time.

The study also found that 2.5% of the mothers whose child had diarrhea reported that they did not take any action for the sickness. This is slightly lower with the findings from west Shoa, Ethiopia (4.3%) and urban slum of Delhi, India (5.8%) [8, 13]. This may be due to the present improvement in awareness about the causes and treatments as well as the positive perceived severity that exist about childhood diarrhea.

In the binary logistic regression model association test was done to identify the determinant factors of mothers’ health seeking behavior for childhood diarrhea. In this study, severity of diarrhea was a significant predictor of health seeking behavior of mothers (P = 0.04). This is consistent with the findings conducted in rural Niger [11].

### Conclusion

This study revealed that nearly three quarter of mothers were health care seekers for if their under-five child had diarrhea. However, a significant number of mothers were treated the childhood diarrhea out of health care settings. Increased thirst, irritability, and decreased fluid intake were the most common symptoms reported. This study also showed that there is a diversity of perception on the causes and treatment options of child hood diarrhea. Severity of diarrhea and blood in stool were the independent determinant factors for health care seeking behavior of mothers in the multivariable analysis.

## Limitations

- The study was employed using interviewer administrated questionnaire that might result social desirability bias.
- Qualitative study is not included in the which was ideal o assess additional factors.

## Abbreviations

AOR: adjusted odds ratio
HEWs: health extension workers
ICCM: integrated community case management
IMNCI: integrated management of neonatal and childhood illness
MDG: millennium development goal
ORS: oral rehydration salt
UNICEF: United Nations Children Emergency Fund
WHO: World Health Organization
